# Dietary Shifts Among the Developmental Stages of the Ectoparasite, *Argulus japonicus* (Crustacea; Branchiura), Mirror Ontogeny as Shown Through Differences in Stable Isotope Ratios of Carbon (δ^13^C) and Nitrogen (δ ^15^N)

**DOI:** 10.1002/ece3.70652

**Published:** 2025-01-09

**Authors:** Beric M. Gilbert, Milen Nachev, Bernd Sures, Annemariè Avenant‐Oldewage

**Affiliations:** ^1^ Department of Zoology University of Johannesburg Johannesburg South Africa; ^2^ Aquatic Ecology and Centre for Water and Environmental Research University of Duisburg‐Essen Essen Germany; ^3^ Research Center One Health Ruhr, Research Alliance Ruhr University of Duisburg‐Essen Essen Germany

**Keywords:** Branchiura, copepodite, feeding, fish lice, larval development, nauplius

## Abstract

Food web architecture and trophic interactions between organisms can be studied using ratios of naturally occurring stable isotopes of carbon (^13^C/^12^C) and nitrogen (^15^N/^14^N). Most studies, however, focused on free‐living organisms, but recently, there has been growing interest in understanding trophic interactions of parasites. The crustacean ectoparasite *Argulus japonicus* is a well‐studied parasite of freshwater teleost fish, which has low host specificity and a cosmopolitan distribution. Little is known about the trophic interactions between various developmental stages of this parasite and its host. This study compares stable isotope ratios of carbon (δ^13^C) and nitrogen (δ^15^N) among developmental stages of *A. japonicus* . It was hypothesised firstly that stable isotopes would vary among the developmental stages of the parasite with differences matching ontogenetic development of the parasite. Secondly, fractionation patterns among developmental stages would relate to different fish tissues and particles, such as algae, ingested by the parasite. Goldfish, *Carassius auratus* , were infected with *A. japonicus,* and different developmental stages of the parasite were isolated and prepared for stable isotope analysis. Differences in stable isotope enrichment correlated with the ontogenetic development of the parasite. In adult parasites, δ^15^N was higher than in the host's tissues, whereas stage two larvae showed the lowest δ^15^N values. Infection by *A. japonicus* alters δ^13^C ratios between infected and uninfected hosts, where the latter group showed lower δ^13^C compared to uninfected hosts. Source contribution comparison showed that algae was not incorporated into the diet of *A. japonicus* and tissues of the host served as the only dietary source of nourishment. These results further suggest that the diet of the parasite is mixed and correlates to the ontogenetic development of *A. japonicus* .

## Graphical abstract

Stable isotope fractionation serves as a fingerprint of nutrients assimilated by organisms. This study aimed to compare fractionation patterns of stable isotope ratios of carbon (δ13C) and nitrogen (δ15N) among various life stages of *A. japonicus* infecting goldfish hosts ( *Carassius auratus* ). Shifts in the ratios of stable nitrogen and carbon isotopes for *A. japonicus* correspond to the development of the mouthparts and digestive cells in the midgut, which limit the parasite feeding on host blood until later developmental stages.

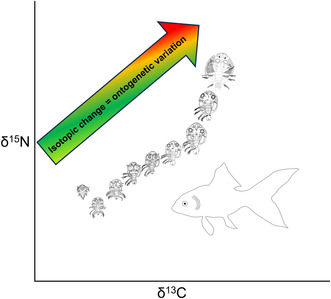

## 1 Introduction

Analysis of stable carbon (δ^13^C) and nitrogen (δ^15^N) isotopes has been implemented to study the food web structure and the diet of various organisms (Minagawa and Wada 1984; Vander Zanden, Cabana, and Rasmussen 1997; Deudero, Pinnegar, and Polunin 2002; Post 2002). Generally, differences in trophic levels are assessed based on specific patterns of enrichment and depletion of stable isotopes of nitrogen and carbon. Consumers have been shown to generally be enriched in ^15^N by a factor of 3.4‰ and 1‰–2‰ for ^13^C, which corresponds to a change of one trophic level in relation to their prey or food source (DeNiro and Epstein 1978; Minagawa and Wada 1984; Vander Zanden, Cabana, and Rasmussen 1997; Kelly 2000). This factor has mostly been applied to understand trophic interactions of free‐living organism in the context of consumer–diet relationships. However, it has also been applied for parasites and their associated hosts as a means of understanding enrichment patterns in relation to feeding strategies of parasites.

Parasites are important components in food web ecology (Marcogliese 2003; Poulin 2010; Nachev et al. 2017; Sabadel, Stumbo, and MacLeod 2019), and several studies have evaluated the fractionation of stable isotopes in parasites compared to their hosts (Nachev et al. 2017; Sures et al. 2019; Gilbert et al. 2020a; Riekenberg et al. 2021). Three main fractionation patterns of stable isotopes between parasites and their host's tissues have been identified. Firstly, some parasite taxa have been shown to become enriched in stable isotopes relative to their hosts similar to predator–prey interactions of free‐living organisms (Iken et al. 2001; O'Grady and Dearing 2006; Nachev et al. 2017; Sures et al. 2019; Jenkins et al. 2020). Secondly, some parasites are depleted in stable isotopes and occupy lower trophic levels compared to their hosts, despite that fact that they utilise nutrients and energy from the host (Deudero, Pinnegar, and Polunin 2002; Navarro et al. 2014; Nachev et al. 2017; Gilbert et al. 2020a, 2020b). Lastly, some parasite taxa show no difference in isotopic levels relative to their host and occupy the same trophic level as their hosts (Pinnegar et al. 2001; Deudero, Pinnegar, and Polunin 2002; Demopoulos and Sikkel 2015; Gilbert et al. 2020a, 2020b). Such patterns can be related to differences in isotopic turnover rates in the host tissues. Parasites feeding on metabolically active tissues with high isotopic turnover will have dietary nitrogen isotope levels that closely match the isotope ratios of the tissues they are consuming (Deudero, Pinnegar, and Polunin 2002; Butterworth, Li, and McKinley 2004; Yohannes et al. 2017; Zhang et al. 2018). The taxonomic position of parasite groups and their related feeding strategies have also been suggested to result in differences in stable isotopes among parasite groups (Nachev et al. 2017; Thieltges et al. 2019; Born‐Torrijos et al. 2023). Nachev et al. (2017) showed that two endoparasites that infect the same host showed significant dissimilarities in their isotope levels, which correlated with the feeding mechanism of both parasite taxa. They found that the nematode, *Eustrongyloides* sp., which actively consumes host tissue, was enriched in stable isotopes compared to the acanthocephalan, *Pomphorhynchus laevis* , which feeds on metabolites released from the host, which are depleted in stable isotopes. Time spent feeding from a single host (Dean, DiBacco, and McKinley 2011; Fritts et al. 2013; Hernández‐Arciga, Herrera, and Morales‐Malacara 2016), the number of host species from which nourishment is derived while feeding (Taccardi, Bricknell, and Byron 2020) and metabolic differences among developmental stages of the same parasite species (Deudero, Pinnegar, and Polunin 2002, Hesse et al. 2022) are other potential sources of variation in stable isotope fractionation among parasites (Thieltges et al. 2019). In their recent meta‐analysis, Thieltges et al. (2019) further indicated that as a result of these variations, parasites deviate from traditional stable isotope fractionation frameworks and as such show differences in trophic discrimination factors (TDFs). When comparing trophic fraction among parasites, they suggested applying a scaled rather than fixed discrimination factor. Most studies have focused on comparing stable isotope levels between adult parasites and their hosts. Few studies have compared differences in stable isotope levels between developmental stages of a single parasite species. This results in a gap in our understanding of parasite trophic interactions, especially considering that some parasite taxa utilise several hosts within their life cycle and, in this way, incorporate isotopic contributions from each of these hosts (reviewed in Born‐Torrijos et al. 2023).

Regarding parasites of fish, studies have focused on an array of taxa, which include various endoparasite and ectoparasite species. Among parasitic crustaceans, most of the available studies on stable isotopes focused on copepods, while only a few of them addressed other crustaceans such as the Branchiura (Table S1). However, copepod and branchiuran parasites have been well studied in terms of pathogenic effects towards their host fish, particularly those species that infect aquaculture fishes (May‐Tec et al. 2022). Among branchiurans, various species of genus *Argulus* were particularly well studied in terms of their biology and pathogenicity (see Neethling and Avenant‐Oldewage 2016; Shinn et al. 2023). The life cycle of *Argulus* spp. is direct and comprises 7–10 developmental stages depending on the species (Tokioka 1936; Shimura 1981; Rushton‐Mellor and Boxshall 1994). The life cycle begins with the hatching of metanauplius larvae from the egg, which are deposited and cemented to hard substrata in the aquatic environment by the female parasite (Tokioka 1936; Shafir and Van As 1986). The metanauplius is a free‐living larva and utilises nourishment from (maternal) yolk deposited in the telolecithal eggs (Tam and Avenant‐Oldewage 2006). After hatching, the constituents of the mouthparts do not change morphologically, but the mouth tube increases in length (Rushton‐Mellor and Boxshall 1994; Lutsch and Avenant‐Oldewage 1995; Walker et al. 2011). Tam and Avenant‐Oldewage (2006) found that the metanauplius stage could not survive for long periods after hatching before seeking a host because of its limited yolk reserves. After infection of a fish host, the metanauplius metamorphoses into the first parasitic larval stage (stage 2) and starts feeding on the host epithelium and mucus and possibly other food particles (Tam and Avenant‐Oldewage 2006) such as algae. All subsequent developmental stages are parasitic, which feed actively on tissues of the host fish.

The diet of *Argulus* spp. consists mainly of blood. However, due to their feeding mechanism, which involves creating lesions in the epidermis of the host in order to access blood vessels, it is likely that other tissues are also opportunistically ingested. Gresty et al. (1993) described the mechanisms of feeding in adult *Argulus japonicus* , whereby the mandibles supported the creation of a lesion in the epidermis of the host to access blood vessels. Various glands connected to the labial spines and preoral spine have been described (Swanepoel and Avenant‐Oldewage 1992). There was debate regarding their role for feeding by *A. japonicus* , whereas most studies attributed their primary role to the release of lytic enzymes and proteins, which prevent blood clotting and also modulate the immune response of the host towards the parasite (Gresty et al. 1993; Walker et al. 2011; AmbuAli et al. 2019). Swanepoel and Avenant‐Oldewage (1992) also demonstrated that most of the glands are connected to ducts associated with the labial and not to the preoral spines. Released enzymes were assumed to contribute to the predigestion of blood and epidermal cells of the host during the feeding (AmbuAli et al. 2019). The crescent‐shaped mandibles, which are equipped with a double row of denticles on the proximal periphery (Paisecki and Avenant‐Oldewage 2008), function to create a lesion in the epidermis and expose blood on which the parasite feeds.

Regarding trophic interaction between *A. japonicus* and its fish host based on stable isotope analysis (SIA), there is no information available to date. All information about the diet of this parasite was derived from studies dealing with the morphology of the mouthparts (Shimura 1983; Gresty et al. 1993; Lutsch and Avenant‐Oldewage 1995; Walker et al. 2011; AmbuAli et al. 2019) as well as the morphology and ultrastructure of the digestive system (Overstreet 1992; Tam and Avenant‐Oldewage 2006, 2009a, 2009b), which cannot allow proper estimation of the primary food sources of the parasite. The morphology and histology of the digestive system have also been found to change among life stages of the parasite (Tam and Avenant‐Oldewage 2006). Tam and Avenant‐Oldewage (2006) provide a detailed description of the morphological and histological changes of the digestive system of *A. japonicus* and confirmed that these changes occur due to specific needs of individual developmental stages to utilise nourishment from different host tissues. They also demonstrated that the cuboidal cells in the anterior midgut of metanauplius of *A. japonicus* contained yolk that serves as the primary source in its early development stages. Walker et al. (2011) confirmed the absence of fish erythrocytes in metanauplius larvae of *A. japonicus* compared to adults, which further supports changes in the different sources of nourishment along with parasite development.

Trophic studies based on SIA among species of the genus *Argulus* and their hosts have only been conducted for *Salmo salar* (Atlantic salmon)—*Argulus foliaceus* host–parasite system (Taccardi, Bricknell, and Byron 2020). The authors found that δ^15^N values of *A. foliaceus* were not significantly higher with respect to its fish host, which was assumed to be related to its low host specificity and potential switch of hosts from different trophic levels. Low host specificity and high pathogenicity are factors that have resulted in much interest in studies of *A. japonicus* and its host fishes, especially under freshwater aquaculture conditions. However, information about potential isotopic shifts during parasite development as a result from change in dietary sources is still lacking. It is hypothesised that variations in isotopic signatures of nitrogen and carbon among the different developmental stages of *A. japonicus* will be due to the utilisation of different food sources by different life stages. Furthermore, given the differences in the ability to feed on different host tissues, it is expected that the consumption of different host tissues contributes to a mixture of dietary sources among developmental stages. In the current study, experiments where goldfish ( *Carassius auratus* ) were deliberately infected with *A. japonicus* were performed to study the host–parasite trophic interaction between the developmental stages of the parasite and its host. Mixing models based on isotopic data were applied for first time for the estimation of dietary contribution of different host tissues to the diet of different development stages of an ectoparasite.

## 2 Materials and Methods

*Argulus japonicus* adults were collected from wild cyprinid fishes (e.g., *Labeobarbus aeneus* , *Labeo capensis* ¸ *Labeo umbratus* ) from the Vaal Dam to establish a laboratory culture. In the laboratory, parasites were maintained on goldfish, *C. auratus* (Linnaeus, 1758) was purchased from a commercial pet shop near the University of Johannesburg and was of a similar size and age (average standard length: 6 ± 1.3 cm). The procedures for maintaining experimental parasite populations on goldfish and collection of tissue samples for SIA were performed following approval from the University of Johannesburg, Faculty of Science Ethics committee (reference number: 2016–5–03). Parasites were collected only from the first laboratory bred generation to ensure that all nutrition was derived from the goldfish. Parasite specimens were collected over a period of 6 months either from live hosts or soon after hosts perished (< 6 h) due to the pathological effects of infection. Frequent removal of parasites was also done from live fish to reduce the impact of high intensities of *A. japonicus* on the survival of the host. Removed parasites were placed in a plastic Petri dish with sufficient aerated water from aquaria until they perished. This was done to ensure the digestive system of the parasite was devoid of food particles, which could have interfered with the stable isotope levels of the parasite. Specimens of *A. japonicus* (*n* = 1591) were then sorted into the various life stages using a stereomicroscope, transferred into 2‐mL microcentrifuge tubes and frozen (−20°C). The developmental stages were identified based on specific morphological features as outlined by Tokioka (1936), Shimura (1981) and Rushton‐Mellor and Boxshall (1994) for *A. japonicus* , *Argulus coregoni* and *A. foliaceus,* respectively.

Host tissues were collected for analysis from both live (*n* = 10) and dead fish (*n* = 31). Live fish were euthanised by first stunning the fish by percussion, severing the spinal cord posterior to the head and then double pithing as required from the South African National Standard: Care and Use of Animals for Scientific Purposes and in line with the approved ethics permit granted by the University of Johannesburg. In the case of fish that died because of infection, fish were frozen soon after death (< 6 h). From both live and dead *C. auratus* hosts, muscle, fins (only epidermis) and skin from the flank (scales removed by scraping) were collected. Blood samples were collected using a 23G needle and heparinised vacutainer only from live hosts before euthanasia. Water samples (50 mL) were collected from the aquaria in plastic centrifuge tubes (Cellstar Tubes, Greiner Bio‐One, Frickenhausen, Germany) and filtered through 0.45‐μm nitrocellulose filter papers to collect algae. As with *A. japonicus* samples, all host tissues and algae samples were frozen at −20°C and then prepared for SIA by first freeze drying the samples with a 1‐2LD plus freeze drier (Martin‐Christ Gefriertrocknungsanlagen, GmbH; Germany). Once dry, host tissues and parasites were homogenised and, where possible, triplicates of each sample were weighed (0.4–0.8 mg) into tin capsules for the analysis of stable isotope ratios of nitrogen (^15^N/^14^N) and carbon (^13^C/^12^C). As a result of the small size of some developmental stages (less than 1000‐μm total length for larval stages) of *A. japonicus*, samples were pooled to a final weight of 0.4–0.8 mg to ensure sufficient sample mass for the analysis. For the analysis of stable isotopes of algae, 3 mg of sample was used. Analysis of samples was performed following procedures described by Nachev et al. (2017) in the C/N mode using an isotope ratio mass spectrometer (IRMS, Isoprime Vision, Elementar, Germany), elemental analyser (EA, Vario ISOTOPE Select, Elementar, Germany). All isotope ratios were reported in δ‐notation as differences in the proportion of isotopes for internal reference standard (acetanilide AcAn) and samples. Isotope values of samples and working standard, acetanilide, were normalised using USGS40 and USGS41 reference materials (International Atomic Energy Agency). Thus, all isotope ratios were reported in δ‐notation with respect to VPDB for carbon and air for nitrogen.

### 2.1 Statistical Analysis

All data were checked for homogeneity and normality using the Shapiro–Wilk test. In cases where samples of different parasite developmental stages were pooled to required weights for analysis, the isotope values were compared between replicates to ensure they did not differ significantly. For trophic level fractionation between *A. japonicus* and its host, the TDF was calculated by subtracting the mean isotopic signatures for δ^13^C and δ^15^N for all host tissues from the stable isotope signature in the developmental stages of *A. japonicus* . Trophic level differences (ΔTL) were assessed for *C. auratus* hosts and *A. japonicus* by using the isotope signatures of the host's muscle tissue and blood with respect to the parasite (Equation 1). Trophic enrichment factor (TEF) was calculated using average values calculated from the literature for other crustacean ectoparasites of fishes (see Table S1). Mean TEF values generated were δ^15^N = 0.36‰ ± 2.56‰ and δ^13^C = −0.32‰ ± 1.88‰. This approach was chosen for TEF determination as there are no published TEF values from controlled studies for other ectoparasites of fishes. Both tissue types were considered to ensure that data were comparable to other studies where only muscle tissue had been compared. Comparison with blood was done as *A. japonicus* is a blood feeding parasite, and therefore, the calculation of ΔTL from muscle tissues alone in this case is poorly applicable to the host–parasite model (Thieltges et al. 2019)

(1) $$∆\mathrm{TL}=\frac{\delta^{15}N_{\text{Argulus}}-\delta^{15}N_{\text{host muscle}}}{\mathrm{TEF}}$$

Isotope differences among the host tissues and *A. japonicus* life stages were evaluated in SPSS v. 28 for Windows (Statistical Package for the Social Sciences, SPSS Inc., USA) using the Kruskal–Wallis test. Pairwise comparisons were performed in cases where the results of the Kruskal–Wallis test were significant. To compare the effect of *A. japonicus* on the isotope signatures of the host, ^15^N and ^13^C levels in fins (epidermal tissue only), muscle and skin of *C. auratus* hosts were compared between infected and uninfected fish. To test the significance of these differences, the Wilcoxon sign rank test was used. Confidence limits for all tests were set at 95% (*p* = 0.05).

Probability estimates of the dietary proportions of isotope sources in the current host–parasite model were obtained using the Bayesian mixing model MixSIAR in R (Stock et al. 2018). Raw and uncorrected values for δ^15^N and δ^13^C for each stage analysed were categorised into broader developmental stages of *A. japonicus* , namely, larvae (stages 2–5), subadult (stages 6–7) and adults for the purposes of the MixSIAR analysis. These were tested against the mean and standard deviation of δ^15^N and δ^13^C in the host tissues, which were the sources of the diet for the parasite. Stage 1 (metanauplii) were not included in the MixSIAR analysis due to insufficient sample mass, which necessitated this stage having to be pooled during analysis. Uninformed priors were applied to the analysis as it was not possible to determine the differences in the amount (weight or volume) of sources consumed by the parasite. Markov chain Monte Carlo parameters selected were as follows: chain length: 3000000, burin‐in: 1500000, thin: 500, number of chains: 3. Following these parameters, convergence of the model was considered suitable following assessment by the Geweke and Gelman–Rubin diagnostics (Stock et al. 2018). As with ∆TL, the mean TEF values calculated from the literature were also used here in the mixing model (δ^15^N = 0.36‰ ± 2.56‰ and δ^13^C = −0.32‰ ± 1.88‰).

## 3 Results

**FIGURE 1:**
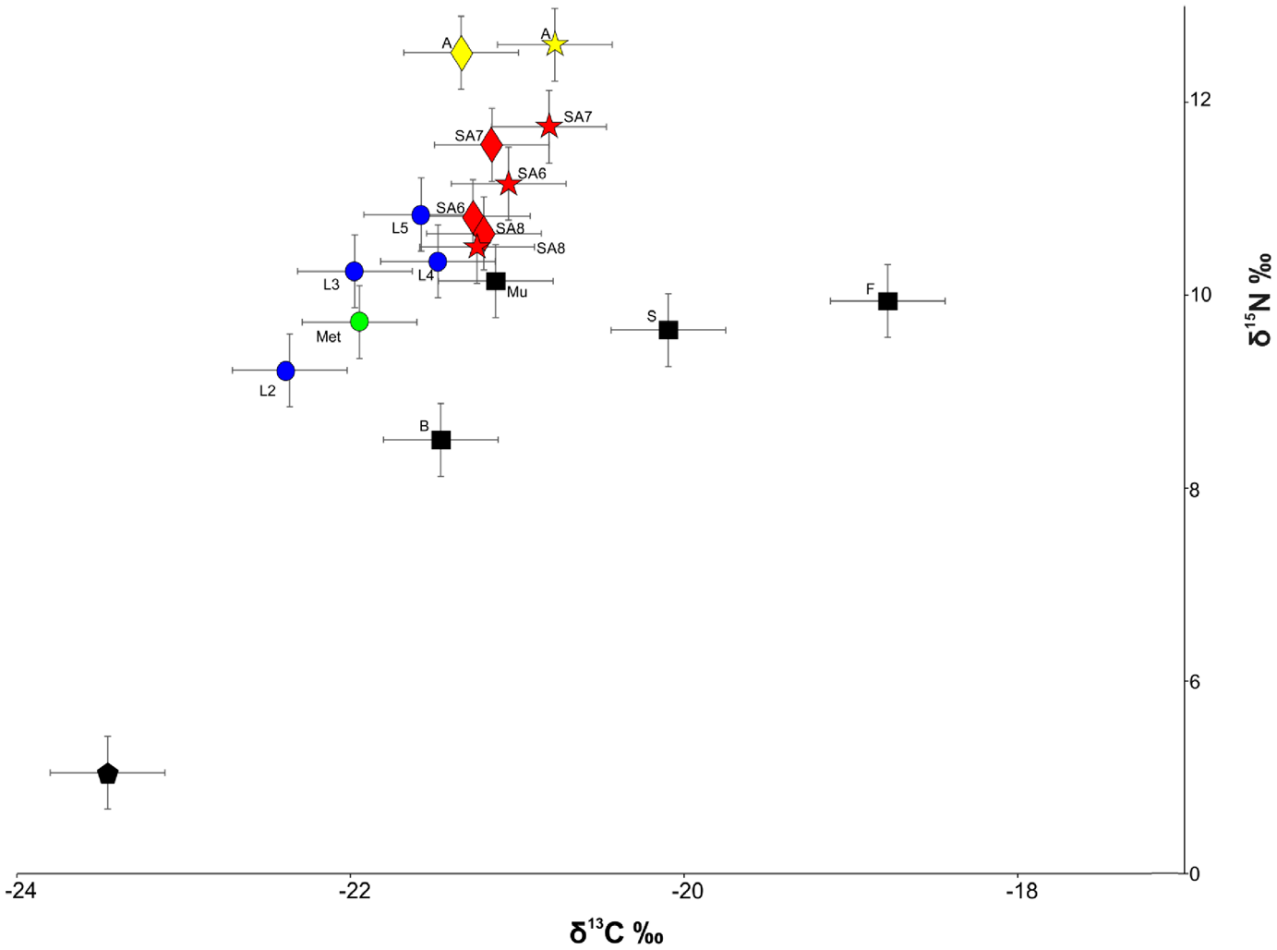
δ^15^N and δ^13^C biplot of mean and standard deviation values for algae, tissues of *Carassius auratus* and developmental stages of *Argulus japonicus* . Black shade in pentagon and squares represent algae (A), blood (B), fin epidermis (F), muscle (Mu) and skin (S) of *C. auratus* . For *A. japonicus* , different colours represent the different developmental groups, with green showing the metanauplii (Met), blue being the larval stages (L2–L5), red represents subadult stages (SA6–SA8) and yellow represents the adults. Symbols have been used to differentiate between immature and mature stages, where sexes could be differentiated, with circles representing immature stages, stars for males and diamond for female parasites.

Stable isotope composition of developmental stages of *A. japonicus* and its potential food source (tissues of fish host and algae) differed significantly (Figure 1 and Table 1). Although δ^15^N among the various tissues of *C. auratus* did not differ significantly (Kruskal–Wallis, *H* = 3.90, df = 3, *p* = 0.272), differences in δ^13^C among host tissues were significant (Kruskal–Wallis, *H* = 30.03, df = 3, *p* < 0.001). The highest δ^15^N ratios were detected for muscle tissues of infected fish (10.2‰ ± 2.17‰), while the lowest were recorded for skin (9.85‰ ± 2.08‰). For δ^13^C, highest ratios were found in muscle (−21.7‰ ± 1.07‰) and lowest in fins (−19.4‰ ± 1.13‰) of infected fish. Comparison between algae and host tissues showed that δ^15^N for algae was lower compared to all host tissues, but δ^13^C ratios were significantly higher compared to the host muscle (pairwise, *x* = −56.6, *p* = 0.01), fin epidermis (pairwise, *x* = −114, *p* < 0.001) and skin (pairwise, *x* = −81.8, *p* < 0.001) tissues. Differences between δ^15^N for algae and host tissues were not significant.

**TABLE 1:** Mean and standard deviation (SD) of δ^15^N and δ^13^C values for tissues of host goldfish ( *Carassius auratus* ) and the crustacean ectoparasite, *Argulus japonicus* . In fish, specific stable isotope levels for infected and uninfected tissues are shown, and for parasites, the mean isotope ratios are shown for different life stages of the parasite.

*Carassius auratus* (goldfish)						
Organ	Infection status	*n*	δ^15^N	SD	δ ^13^C	SD
Fin epidermis	Uninfected	12	9.47	1.18	−16.81	1.37
	Infected	21	10.09	1.84	−19.41	1.13
Muscle	Uninfected	12	9.88	1.29	−19.25	1.69
	Infected	21	10.23	2.17	−21.73	1.07
Skin	Uninfected	12	8.99	0.94	−18.51	2.00
	Infected	21	9.85	2.08	−20.62	1.25
Blood	Uninfected	12	8.50	0.72	−21.46	0.79

*Argulus japonicus*					
Stage	*n*	δ^15^N	SD	δ ^13^C	SD
Adult female	10	12.52	1.42	−21.34	0.52
Adult male	7	12.60	1.07	−20.78	0.57
Subadult male stage 8	1	10.50		−21.24	
Subadult female stage 8	3	10.64	0.58	−21.20	0.38
Subadult male stage 7	4	11.75	0.74	−20.81	0.34
Subadult female stage 7	4	11.56	0.60	−21.15	0.67
Subadult female stage 6	3	10.82	0.20	−21.27	0.30
Subadult male stage 6	3	11.16	0.12	−21.05	0.21
Juvenile stage 5	1	10.84		−21.58	
Juvenile stage 4	2	10.35	0.18	−21.48	0.50
Juvenile stage 3	3	10.25	0.18	−21.97	0.20
Juvenile stage 2	1	9.22		−22.36	
Metanauplii	1	9.72		−21.95	

Variation in the stable isotope composition among the developmental stages of *A. japonicus* (Figure 1) showed a progressive enrichment in ^15^N from the metanauplius toward adult stages, whereas the differences in ^15^N between developmental stages were significant (Kruskal–Wallis, *H* = 25.4, df = 8, *p* = 0.001). In contrast, δ^13^C (Kruskal–Wallis, *H* = 9.97, df = 8, *p* = 0.267) showed no significant difference among developmental stages of the parasite. Adult parasites showed the highest enrichment in ^15^N, and the lowest was obtained for stage 2 larvae. Metanauplii (stage 1) were enriched in ^15^N but depleted in ^13^C compared with stage 2 larvae.

In comparison to the host tissues, stage 7 (subadults) and adults of *A. japonicus* were significantly higher in δ^15^N compared to the fin epidermis, muscle, skin and blood of the host (pairwise, in all cases *p* ≤ 0.05). The signatures of δ^15^N stage 6 (subadults) and host blood were also significantly different (pairwise, *x* = −63.5, *p* = 0.008). Only δ^13^C values of skin were significantly higher than in most of the developmental stages of the parasite. Developmental stages 5 and 8 did not differ significantly in δ^13^C compared to the fin epidermis. All other comparisons for δ^13^C between fish tissues and parasite were not significant (pairwise, *p* > 0.05 in all comparisons).

TDF calculated between developmental stages of *A. japonicus* and the tissues of *C. auratus* hosts are shown in Table 2. Compared with all host tissues, adult parasites consistently showed higher enrichment in the nitrogen, whereas a difference of 3.2‰ and 3.1‰ in adult male and female parasites with respect to all tissues was accounted. Based on ∆^15^N values of all larval stages and host tissue combinations, in all cases, larval stages showed in general a slight enrichment in ^15^N compared to host tissues. Only for the second larval stage, the ∆^15^N values were lower (−0.17‰) compared to host tissues, showing that the stage is depleted in ^15^N. In all instances, larval stages had TDF values less than or equal to 1‰ for ∆^15^N showing that parasites shared similar ^15^N levels with host tissues. TDF values for ∆^15^N between blood and parasites showed differences between 2‰ and 4‰ from stage 5 larvae to adults. In the case of ∆^13^C values between parasites and host tissues, parasites were depleted in ^13^C compared to host tissues. Only skin and fin epidermis showed higher values (indicating enrichment) for all parasite stages than host tissues. For blood—larvae comparisons, ∆^13^C showed higher values in parasites than in host blood.

**TABLE 2:** Trophic discrimination factors for ∆^15^N and ∆^13^C stable isotopes in the different developmental stages of *Argulus japonicus* compared to the tissues of the host fish, *Carassius auratus* .

	Developmental stage	∆^15^N (all tissues)	∆^13^C (all tissues)	∆^15^N (muscle)	∆^13^C (muscle)	∆^15^N (blood)	∆^13^C (blood)	∆^15^N (skin)	∆^13^C (skin)	∆^15^N (fins)	∆^13^C (fins)
Larva	Metanauplius	0.331	−1.15	0.622	−0.218	1.22	−0.487	−0.149	−1.35	−0.369	−2.54
	Stage 2	−0.168	−1.57	0.123	−0.636	0.721	−0.905	−0.648	−1.77	−0.868	−2.96
	Stage 3	0.859	−1.18	1.15	−0.246	1.75	−0.515	0.378	−1.38	0.159	−2.57
	Stage 4	0.961	−0.679	1.25	0.253	1.85	−0.016	0.481	−0.883	0.261	−2.07
	Stage 5	1.45	−0.780	1.74	0.152	2.34	−0.117	0.966	−0.984	0.746	−2.17
Subadult	Stage 6 ♂	1.77	−0.256	2.06	0.676	2.66	0.406	1.29	−0.460	1.07	−1.65
	Stage 6 ♀	1.43	−0.470	1.72	0.462	2.32	0.193	0.950	−0.674	0.730	−1.86
	Stage 7 ♂	2.36	−0.013	2.65	0.919	3.25	0.649	1.88	−0.217	1.66	−1.40
	Stage 7 ♀	2.17	−0.356	2.46	0.576	3.06	0.307	1.69	−0.560	1.47	−1.75
	Stage 8 ♂	1.11	−0.445	1.40	0.487	2.00	0.218	0.630	−0.649	0.411	−1.84
	Stage 8 ♀	1.25	−0.404	1.54	0.528	2.14	0.259	0.770	−0.608	0.550	−1.79
Adult	Adult ♂	3.21	0.021	3.50	0.953	4.10	0.684	2.73	−0.183	2.51	−1.37
	Adult ♀	3.13	−0.541	3.42	0.391	4.01	0.122	2.64	−0.745	2.43	−1.93

**FIGURE 2:**
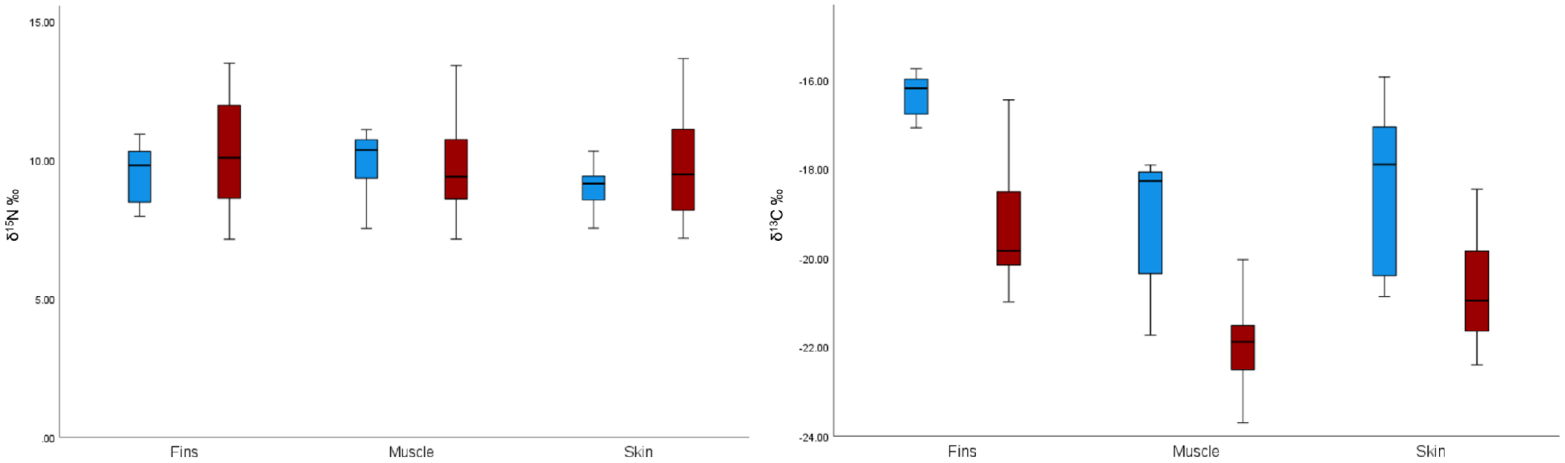
Bar charts showing differences in δ^15^N and δ^13^C for tissues of uninfected (blue bars) and infected (red bars) *Carassius auratus* . Error bars represent 95% confidence intervals.

**FIGURE 3:**
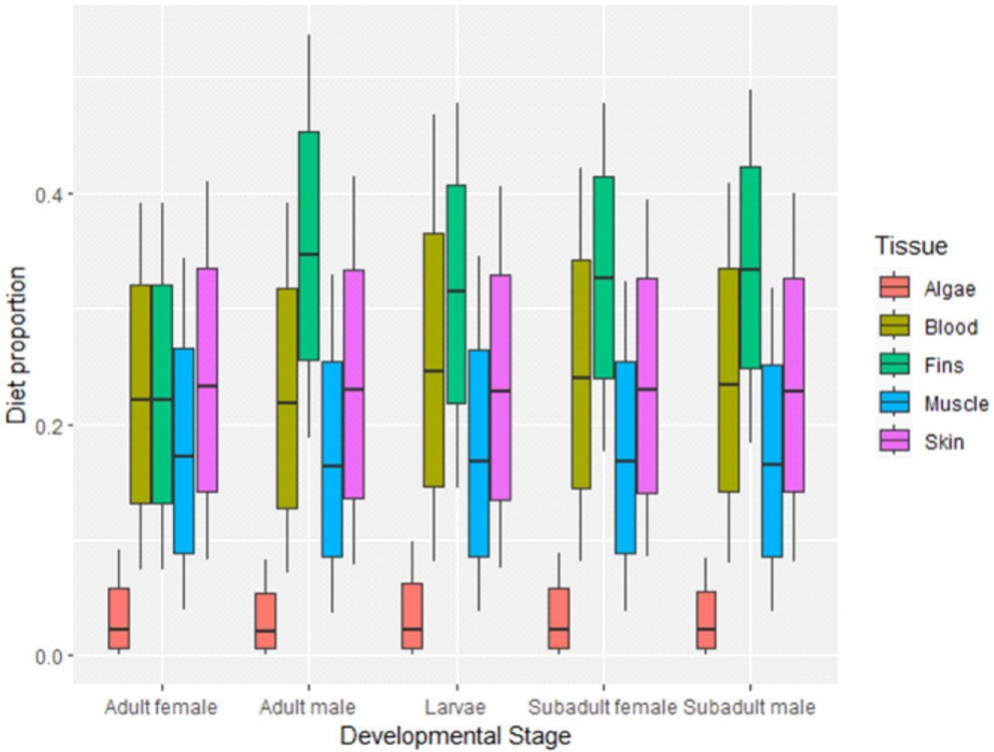
Box plot showing summary data for the dietary proportions of *Carassius auratus* host tissues (muscle, fins, blood and skin) and algae for adult males and females, subadult males and females and larvae of *Argulus japonicus* . The key shows the different tissues that were analysed as dietary sources. Error bars represent 95% confidence intervals.

Infection status of *C. auratus* affected only δ^13^C values, whereas uninfected fish showed significantly lower ^13^C enrichment for all tissues in comparison to infected ones (pairwise, *p* < 0.05 in all comparisons; also see Figure 2). According to the output of mixing modes (MixSIAR), fin epidermal tissue accounted for the highest contribution to the diet of the parasite in all parasitic life stages of *A. japonicus* . Overall, this tissue type accounted for 33.2% of the diet of the parasite (Figure 3). This was followed by blood and skin, which accounted overall for approximately 23.5% and 23.3%, respectively, of the overall diet for all developmental stages. Muscle accounted for the lowest contribution in the diet of the parasite, and algae did not account for any dietary contributions in the developmental stages of the parasite. For adult parasites, skin showed the second highest contribution to diet of males and females, followed by the blood. In subadult male and females, and all larvae, the opposite trend was observed, where after fin epidermis, blood exhibited the second highest contribution, followed by the skin. Due to the small size of metanauplius stages, samples collected were pooled for analysis and this resulted in a single data point (*n* = 1), which was used as indicative isotopic values for this life stage, but these data were not considered in the MixSIAR analysis.

## 4 Discussion

Results of the current study showed differences in the ratios of stable isotopes of carbon and nitrogen among developmental stages of *A. japonicus,* which mirrored the ontogeny from larvae to adults. The adult parasites showed the highest enrichment in ^15^N, while for the second larval stage, the lowest one was observed. This finding aligns well with the current knowledge about the development of stage 2 larvae (the first parasitic stage) from the metanauplius stage. Once the metanauplius metamorphoses into the second larval stage (Rushton‐Mellor and Boxshall 1994; Lutsch and Avenant‐Oldewage 1995), it has depleted all yolk reserves it received from the adult female parasite and needs to start feeding on host tissues (Hakalahti 2005). The decrease in δ^15^N from the metanauplius to stage 2 larvae coincided with transition in nourishment from yolk to feeding on a host. From this point, values of ^15^N showed gradual enrichment along the successive developmental stages, which corresponded to the ontogeny of the parasite. The gradual increase in δ^15^N further relates to the longer time the parasite spends feeding on host fish tissues as it matures. Enrichment in ^15^N of adult parasites compared to larval stages was similarly observed by Demopoulos and Sikkel (2015) for gnathiid isopods infecting reef fishes, which similarly feed on blood of the host. They attributed this fractionation pattern to assimilation and digestion of blood and subsequent metamorphosis among developmental stages. In a parasitic copepod, *Lamproglena clariae*, Gilbert et al. (2020a) showed that eggs in the egg strings of the adult female parasites had a higher δ^15^N ratio than the adult parasites. This was related to the fact that the larvae developing in the egg received all nourishment needed for development from the adult parasite.

Changes in stable isotope ratios between the various developmental stages can be related to the ability of the parasite to feed on their host. Morphologically, all functional components of the mouthparts are present in the metanauplius (Rushton‐Mellor and Boxshall 1994; Lutsch and Avenant‐Oldewage 1995). However, despite this, the second larval stage is unable to feed on the blood of the host. This has been shown by Tam and Avenant‐Oldewage (2006) where the mandibles in the first larval stage are of insufficient size to penetrate the epidermis and gain access to blood vessels of the host. Following this, Walker et al. (2011) confirmed that the larvae of *A. japonicus* are unable to feed on whole blood due to the diameter of the buccal opening being smaller than the size of erythrocyte, which limits the entry of blood components into the buccal cavity and digestive system. The latter authors confirmed this by demonstrating a lack of host erythrocytes in the digestive system of larvae compared to adults, where blood cells were detected. Along with this, it was reported that the cells of the anterior midgut of metanauplius larvae were filled with yolk (Tam and Avenant‐Oldewage 2006). Results of the current study further confirmed that the diet of *A. japonicus* comprises a mixture of host tissues. This can be explained through the feeding mechanisms of the parasite, whereby paired mandibles are used to mechanically strip the epidermis and create a lesion in the skin (Gresty et al. 1993; Paisecki and Avenant‐Oldewage 2008). Along with this, lytic enzymes released from glands in the labial spines and preoral spine (Swanepoel and Avenant‐Oldewage 1992) are likely to assist the predigestion of host tissues (AmbuAli et al. 2019), the prevention of blood clotting and the modulation of the immune response (Gresty et al. 1993; Walker et al. 2011; AmbuAli et al. 2019). As a result, various tissues might be utilised as a food source and thus contribute to a mixing of stable isotope of different dietary sources.

In terms of the effect of infection on the stable isotope fractionation in parasitised hosts, a few studies have reported on this aspect (reviewed in Born‐Torrijos et al. 2023). In the present study, infection by *A. japonicus* resulted in significant differences in carbon isotope levels between infected and uninfected goldfish hosts, whereas parasitised fish showed significantly lower δ^13^C values than unparasitised hosts. For δ^15^N values, infection status had little effect on fractionation of this isotope between parasitised and unparasitised fish. Pulkkinen, Aalto, and Nykänen (2016) found that *Daphnia magna* infected with the microsporidian *Glugoides intestinalis* had increased δ ^13^C compared to uninfected hosts. They related this to the effect of the microsporidian parasites on the gut epithelial cells, suggesting that higher δ^13^C values in infected *D. magna* were related to the increased excretion of carbon in the gut, which would contribute to ^13^C enrichment in infected animals. In the present study, once *C. auratus* hosts became infected by *A. japonicus,* they fed less frequently on commercial fish food provided. However, unlike *G. intestinalis* , *A. japonicus* is an ectoparasite and only infects the outer body surface and fins of its host fish and, therefore, it has little direct effect on the functioning of the intestine in the host and enrichment in ^13^C but could have been affected by reduced feeding by infected hosts. Factors such as stress, tissue‐specific turnover rates, metabolism and lipid content of tissues are known to affect the fractionation of carbon in organisms (Hesslein, Hallard, and Ramlal 1993; Buchheister and Latour 2010; Carleton and del Rio 2010; Mont'Alverne 2016; Martino, Doubleday, and Gillanders 2019). Infection, which leads to stress in the fish host, may result in changes in the metabolic rate of the fish and by extension the fractionation of the carbon isotope. Kabata (1985) showed that hosts that became infected with *Argulus* sp. suffered appetite suppression, which resulted in a reduced growth rate and appeared emaciated. In a report by Taylor (2005), it was noted that the reduced angling success of fishes infected by *Argulus* spp. could be related to a reduction in feeding due to a loss of appetite once hosts became infected by the parasite. A reduced consumption of food by infected goldfish in the present study may relate to the effect of starvation on lipid reserves, which would become enriched overtime and would have affected δ^13^C. This would occur due to a change in carbon metabolism. Similarly, Born‐Torrijos et al. (2023) recently pointed out that the infection of hosts by parasites would affect stable isotope signatures of host tissues, which could further compound and influence estimations of the trophic level of the host. However, this aspects deserve further investigations to address the mechanisms leading to such differences.

Trophic level differences (∆TL) indicate that adults of *A. japonicus* further behave as (micro) predators with ∆δ^15^N of approximately 3‰ compared to tissue isotope levels of the host. From stage 3 larvae onwards to adults of *A. japonicus* , the increase in δ^15^N could parallel with progressive feeding on the host. The feeding biology of the parasite, therefore, corresponds to a gradual enrichment in ^15^N between successive developmental stages. This can be further related to the phrase ‘You are what you eat’, which, in this case, corresponds to feeding duration and the type of tissue being consumed. This possibly resulted in changes in the isotopic composition of various developmental stages of the parasite. On the other hand, and contrary to our results for *A. japonicus* , Taccardi, Bricknell, and Byron (2020) found that *A. foliaceus* was not enriched in ^15^N compared to its host fish, *S. salar* . They attributed this to the low host specificity of *A. foliaceus* and suggested that feeding from different hosts, which occupy different trophic levels, could have resulted in unclear enrichment patterns as the parasite might derive nutrition from multiple fish species during its ontogeny. In the present study, only one host species was infected and studied; therefore, no contributing effects from the host can be expected. It was clear from the present results that adults of *A. japonicus* were significantly higher in δ^15^N compared to the fish host. Taccardi, Bricknell, and Byron (2020) also described the enrichment of ^15^N in the copepod parasite, *Lepeophtheirus salmonis* , which only feeds on one individual host specimen. In this case, they found higher δ^15^N for adult *L. salmonis* when compared to the host. The enrichment in ^15^N of adult *A. japonicus* relative to the host muscle tissue corresponds to fractionation patterns observed for other haemophagous ectoparasites where comparisons were made between δ^15^N of the parasite and muscle tissue of the host (Boag et al. 1998; Doucett, Giberson, and Power 1999; Voigt and Kelm 2006; Schmidt et al. 2011; Sures et al. 2019). However, compared to studies on other ectoparasitic crustaceans and their associated hosts, the fractionation patterns are not consistent (Summary of the literature for fractionation of stable isotopes of nitrogen and carbon among various host–crustacean parasite associations Table S1). In most instances, the parasites showed either lower or similar δ^15^N values to the host, even though in most cases, the source of nutrition is similar among parasite taxa. Some reasons can be provided for this high variability such as the duration of feeding and residence on a host, as well as the turnover rates of the tissues being fed on (Taccardi, Bricknell, and Byron 2020). This has been shown to particularly influence ^15^N levels in organisms (Doi, Akamatsu, and González 2017). The current study is based on controlled experiments where infected and uninfected hosts were fed at a constant rate, unlike other studies, which have focused on wild captured host–parasite systems. During experiments, infected fish showed a reduction in feeding, which resulted in the enrichment of ^13^C. It has also been shown that the effects of starvation are variable, and currently there is a lack of consensus regarding this in stable isotope ecology (Haubert et al. 2005; del Rio et al. 2009). Therefore, future studies using this approach as a means of excluding host tissue isotope levels in whole organisms used for SIA studies should assess the effects of starvation on organisms by comparing fasted and nonfasted specimens.

The present study is the first to apply MixSIAR to estimate source contributions within the diet of a fish ectoparasite. The mixing model showed that overall, there was little variation in the source contributions of different host tissues. However, there was a higher contribution by the fin epidermis followed by blood of the host fish, *C. auratus* , to the dietary isotope levels of the developmental stages of *A. japonicus* . These results corroborate suggestions of a mixed diet for *A. japonicus* (Tam and Avenant‐Oldewage 2006; Walker et al. 2011). Walker et al. (2011) outlined that there has been much controversy surrounding the diet of *A. japonicus* with suggestions of dietary sources ranging from fish mucous (LaMarre and Cochran 1992) to blood (Poulin and FitzGerald 1988; Mikheev, Valtonen, and Rintamäki‐Kinnunen 1998; Mikheev et al. 2000; Pasternak et al. 2000) where the parasite could even selectively ingest blood serum and not whole blood (see Gresty et al. 1993). The mixing of dietary sources for *A. japonicus* recorded in the present study can be related to the differences in morphology of feeding structures of the parasite. Although the main source of nutrition is probably blood, the parasite gains access to the blood vessels of the fish by first physically damaging and removing the epidermis and the underlaying dermis of the skin on the body and fins. In doing this, the parasites create a lesion where blood forms pools and the parasite ingests blood by telmophagy aided by the vacuum formed by the circular muscles around the oesophagus. Gresty et al. (1993) described the feeding process of *A. japonicus* , whereby the mouth tube becomes erected by adductor muscles and in doing this the mandibles, which are used to dislodge host tissue and form a lesion, are brought into contact with host epidermis. Gresty et al. (1993) further indicated that although *A. japonicus* shows a grazing feeding behaviour, the formation of clearly delimited lesions created by the parasite indicates that it spends time feeding in the same place. Paisecki and Avenant‐Oldewage (2008) reported haemorrhagic areas on the epidermis where the parasite occasionally ingested dermis and stratum spongiosum along with blood. Therefore, while creating lesions in order to gain access to the blood of the host, other tissues at the feeding site are similarly ingested. In terms of the dietary mixing of stable isotopes relating to feeding on specific tissues by parasites, Sabadel et al. (2022) also showed that when the parasitic barnacle *Anelasma squalicola* attached to the eye of the Southern lanternshark ( *Etmopterus granulosus* ) host, δ^15^N ratios were greater than when the barnacles were attached to other tissues.

It is important to note that while the MixSIAR results presented here indicate support for host tissues as a dietary source for the various life stages of *A. japonicus* , these findings should be interpreted in light of the lack of significant differences in isotope levels of host tissues. As a result of the lack of significant difference among host tissue stable isotope levels, it is likely that the model cannot accurately predict specific contributions of individual host tissues (Fry 2013; Phillips et al. 2014; Stock et al. 2018). This therefore highlights the need for mixing models, which can be specifically applied for parasite models, where nourishment from single host sources is obtained and which potentially incorporate different tissues.

The greater contribution of blood to the diet of the parasite may be due to the blood of *C. auratus* in the present study having lower δ^15^N compared to fin epidermis. In the case of including algae in the current analysis, Tam and Avenant‐Oldewage (2006) suggested that based on the morphology, the cuboidal cells of the epithelium of the posterior midgut of larval stages were likely insufficient to digest blood (these cells change to papillated columnar cells in adults). They suggested that these cells might be sufficient in the digestion and absorption of mucus and other particles that could supplement the diet in earlier developmental stages, such as stage 2 larvae. This is further supported by the fact that the mandibles and mouth of earlier ontogenetic stages are too small to access blood vessels and facilitate consumption of whole blood (Tam and Avenant‐Oldewage 2006; Walker et al. 2011). Due to these morphological limitations in early larval stages, it is likely that nourishment other than host blood is consumed to sustain the larval stage; however, from the results of this study, it is clear that algae is not consumed as an alternative food source.

## 5 Conclusion

During the present study, it was hypothesised that based on differences in ontogenetic development, fractionation of ^13^C and ^15^N would differ based on variations in time spent feeding on the blood of cyprinid host fish, and ultimately, this would correlate with the ontogenetic sequence of the *A. japonicus* . From the results, it was clear that δ^15^N and δ^13^C ratios mirrored the ontogenetic development of the parasite, with lower isotope levels in earlier developmental stages and a gradual increase to highest levels in adult stages. The gradual increase in isotope levels in *A. japonicus* could further relate to the length of time the parasite has spent feeding on host tissues from larval stages through to adult stages. It was also hypothesised that non‐host particles, such as algae, could contribute to isotope levels in earlier developmental stages. With regard to the supplementation of the diet of the parasite by sources other than those of the host origin, this was assessed by including algae as an external/non‐host source. It was shown with the use of the mixing model MixSIAR that algae are not included in the diet of any of the life stages of the parasite. However, given the fact that particles in the water column may become lodged in the mucus of the fish that covers the skin, these may be simultaneously ingested by parasites while feeding on host tissues. Consumption would be achieved via the sweeping movements and sharp spines on the mandibles, which aid in removing the epidermis to expose blood vessels below. Results of the present study not only demonstrate the usefulness of stable isotopes in differentiating trophic variation between hosts and parasites but can also be used as a useful technique for studying trophic changes in relation to the ontogeny of organisms. Similar studies would be useful for studying other crustacean parasites, such as selected parasitic Copepoda, which like *Argulus* have several developmental stages in the life cycle. In addition to differences between life stages, some parasitic copepods further differ in that only females become parasitic after copulation, but males remain free‐living. In such a case, stable isotope analysis and the use of mixing models can be useful for understanding variations in the diet of these organisms during ontogeny and between sexes.

## Author Contributions

**Beric M. Gilbert:** conceptualization (equal), data curation (equal), formal analysis (lead), investigation (lead), validation (equal), visualization (equal), writing – original draft (equal), writing – review and editing (equal). **Milen Nachev:** formal analysis (equal), investigation (equal), methodology (equal), validation (equal), writing – review and editing (equal). **Bernd Sures:** formal analysis (equal), investigation (equal), writing – review and editing (equal). **Annemariè Avenant‐Oldewage:** conceptualization (equal), formal analysis (equal), funding acquisition (equal), investigation (equal), project administration (equal), resources (equal), supervision (equal), validation (supporting), visualization (supporting), writing – original draft (equal), writing – review and editing (equal).

## Conflicts of Interest

The authors declare no conflicts of interest.

## Supporting information


**Table S1.** Summary of ^15^N fractionation among parasitic crustaceans infecting aquatic hosts. TEF for host–parasite systems presented was calculated based on δ^15^N for host muscle tissues. Bolded values for TEF indicate where parasites showed enrichment in ^15^N compared to the host.

## Data Availability

All raw data are available from the first author upon reasonable request.
