# Supplementary material for: Dietary Shifts Among the Developmental Stages of the Ectoparasite, Argulus japonicus (Crustacea; Branchiura), Mirror Ontogeny as Shown Through Differences in Stable Isotope Ratios of Carbon (δ13C) and Nitrogen (δ 15N)
Source: Ecol Evol. 2025 Jan 9;15(1):e70652. doi: 10.1002/ece3.70652 (PMC11717899; doi:10.1002/ece3.70652)
Supplement: Supplementary file 1 — Table S1. Summary of 15N fractionation among parasitic crustaceans infecting aquatic hosts. TEF for host–parasite systems presented was calculated based on δ15N for host muscle tissues. Bolded values for TEF indicate where parasites showed enrichment in 15N compared to the host. [file ECE3-15-e70652-s001.docx]

Table S1. Summary of ^15^N fractionation among parasitic crustaceans infecting aquatic hosts. TEF for host-parasite systems presented was calculated based on δ^15^N for host muscle tissue. Bolded values for TEF indicate where parasites showed enrichment in ^15^N compared to the host.

| Reference | Parasite | Order | Host | Developmental stage | δ^15^N Host | δ^15^N Parasite | ∆^15^N/TEF  (parasite-host) | Nutrition source (parasite) | Parasite microhabitat |
| --- | --- | --- | --- | --- | --- | --- | --- | --- | --- |
| Gresty and Quarmby 1991 | *Mytilicola intestinalis* | Cyclopoida | *Mytilis edulis* | Adult | 4.50 | 7.30 | **2.80** | Blood | Intestine |
| Iken et al. 2001 | Copepoda | NA | *Coryphaenoides armatus* | Adult | 15.02 | 17.72 | **2.70** | Blood | Gills |
| Pinnegar et al. 2001 | *Lernaeocera branchialis* | Siphonostomatoida | *Platichthys flesus* | Adult | 13.80 | 12.99 | -0.81 | Blood | Gills |
|  | *Anilocra physodes* | Isopoda | *Boops boops* | Adult | 7.44 | 7.14 | -0.30 |  | Skin |
| Deudero et al. 2002 | *Lernaeenicus sprattae* | Siphonostomatoida | *Sprattus sprattus* | Adult | 12.88 | 13.48 | 0.60 | Blood | Eye |
|  | *Clavella adunca* |  | *Gadus morhua* | Adult | 13.82 | 12.53 | -1.29 | Blood and gill tissue | Gill |
|  | *Lernaeocera branchialis* |  |  | Adult | 13.82 | 13.00 | -0.82 | Blood | Gill and heart |
|  | *Lernaeocera branchialis* |  | *Melanogrammus aeglefinus* | Adult | 15.64 | 16.74 | **1.10** | Blood | Gill |
|  | *Clavella adunca* |  | *Merlangius merlangius* | Adult | 15.64 | 11.41 | -4.23 | Blood and gill tissue | Gill |
|  | *Lernaeocera branchialis* |  |  | Adult | 15.64 | 13.03 | -2.61 | Blood | Gill and heart |
|  | Copepoda | NA |  | Adult | 15.64 | 9.82 | -5.82 | NA | Gill |
|  | *Lepeophtheirus pectoralis* | Siphonostomatoida | *Platichthys flesus* | Adult | 8.90 | 8.68 | -0.22 | Mucus, skin and blood | Skin |
| Baud et al. 2004 | *Neoergasilus japonicus* | Cyclopoida | *Perca fluviatilis* | Adult | 12.10 | 15.80 | **3.70** | Fin epidermis | Fins |
| Xu et al. 2007 | *Ichthyoxenus japonensis* | Isopoda | *Carassius auratus auratus* | Adult | 9.80 | 9.20 | -0.60 |  | Peritoneal cavity |
| Demopoulos and Sikkel 2015 | *Gnathia marleyi* | Isopoda | *Stegastes diencaeus* | NA | 7.40 | 6.60 | -0.80 | Blood | Skin |
|  | *Gnathia marleyi* |  | *Haemulon flavolineatum* | NA | 7.90 | 6.90 | -1.00 | Blood | Skin |
|  | *Anilocra haemuli* |  |  | Adult | 7.90 | 8.80 | 0.90 | Blood | Skin |
|  | *Anilocra haemuli* |  |  | Juvenile | 7.90 | 8.70 | 0.80 | Blood | Skin |
|  | *Anilocra holocentri* |  | *Holocentrus adscensionis* | Adult | 7.90 | 7.70 | -0.20 | Blood | Skin |
|  | *Anilocra holocentri* |  |  | Juvenile | 7.90 | 7.80 | -0.10 | Blood | Skin |
| Goedknegt et al. 2018 | *Mytilicola orientalis* | Cyclopoida | *Mytilus edulis* | Adult | 11.42 | 12.64 | **1.22** | Host tissue and gut contents | Intestine |
| Gilbert et al. 2020a | *Lamproglena clariae* | Cyclopoida | *Clarias gariepinus* | Adult | 16.94 | 17.68 | 0.74 | Blood | Gills |
|  |  |  |  | Eggs | 17.68* | 19.56 | **1.88** | Yolk | Yolk in egg |
| Jenkins et al. 2020 | *Caligus atromaculatus* | Siphonostomatoida | *Acanthurus coeruleus* | Adult | 6.30 | 7.40 | **2.80** | N/A | Skin |
|  |  |  | *Acanthurus bahianus* | Adult | 5.80 | 6.90 | **2.80** | N/A | Skin |
| Taccardi et al. 2020 | *Argulus foliaceus* | Arguloida | *Salmo salar* | Adult | 12.53 | 12.80 | 0.21 | Blood | Skin and fins |
|  | *Lepeophtheirus salmonis* | Siphonostomatoida |  | Adult | 14.98 | 12.53 | **2.45** | Blood and skin | Skin |

NA – sufficient details relevant to the information present were not included in the associated publication.

* Adult parasite treated as “host” in the case of eggs as all nourishment for developmental stages within the egg is derived from yolk/vitellogenin which comes from the adult female.

References

Baud, A. et al. 2004. Seasonal variability in the gut ultrastructure of the parasitic copepod *Neoergasilus japonicus* (Copepoda, Poecilostomatoida). - Can. J. Zool. 82: 1655–1666.

Goedknegt, M. A. et al. 2018. Trophic relationship between the invasive parasitic copepod *Mytilicola orientalis* and its native blue mussel (*Mytilus edulis*) host. - Parasitology 145: 814–821.

Gretsy, K. A. and Quarmby, C. 1991. The trophic level of *Mytilicola intestinalis* Steuer (Copepoda: Poecilostomatoida) in *Mytilus edulis* L., as determined from stable isotope analysis. - Bull. Plankt. Soc. Japan: 363–371.

Xu, J. et al. 2007. Trophic relationship between the parasitic isopod *Ichthyoxenus japonensis* and the fish *Carassius auratus auratus* as revealed by stable isotopes. - J. Freshw. Ecol. 22: 333–338.
